# Nurse-led care versus neurologist-led care for long-term outcomes of patients who underwent craniotomy in traumatic brain injuries: an efficacy analysis

**DOI:** 10.3389/fneur.2024.1382696

**Published:** 2024-07-26

**Authors:** Jingjing Wang, Zhiping Wu, Shufang Shi, Jiangyan Ren, Xiaojia Ren

**Affiliations:** ^1^Department of Neurosurgery, Shanxi Bethune Hospital, Shanxi Academy of Medical Sciences, Third Hospital of Shanxi Medical University, Tongji Shanxi Hospital, Taiyuan, China; ^2^Teaching and Clinical Skills, Shanxi Bethune Hospital, Shanxi Academy of Medical Sciences, Third Hospital of Shanxi Medical University, Tongji Shanxi Hospital, Taiyuan, China

**Keywords:** anxiety, burden on caregivers, craniotomy, cognitive conditions, daily living activity, depression, nurse, neurologist

## Abstract

**Background:**

After craniotomy, patients require rehabilitation efforts for the recovery process, and neurologists are mostly engaged for that (in the management of post-craniotomy complications). However, neurologists are not always available for care after neurosurgery during follow-up (situation of our institute). The objectives of the study were to compare the effects of two different types of care (nurse-led and neurologist-led) on various long-term outcomes in patients who have undergone craniotomy due to traumatic brain injuries.

**Methods:**

Electronic medical records of patients (aged ≥18 years) who underwent craniotomy for traumatic brain injuries and their caregivers were extracted and retrospectively reviewed. Patients received nurse-led care (NL cohort, *n* = 109) or neurologist-led care (GL cohort, *n* = 121) for 6 months after craniotomy.

**Results:**

Before the nurse-or neurologist-led care (BC), all patients had activities of daily living (ADL) ≤ 11, ≤ 50 quality of life (QoL), and 69% of patients had definitive anxiety, 87% of patients had definitive depression, and all caregivers had Zarit Burden interview scores ≥50. Nurse-led post-surgical care was associated with improved ADL and QoL, relieved anxiety and depression of patients, relieved the burden on caregivers, and the higher overall satisfaction of patients and their caregivers after 6-months of care (AC) as compared to their BC condition (*p* < 0.05) and also compared to those of patients in the GL cohort under AC condition (*p* < 0.01). Patients in the GL cohort reported pressure sores (*p* = 0.0211) and dizziness [15 (12%) vs. 5 (5%)] after craniotomy during follow-up than those in the NL cohort.

**Conclusion:**

ADL, QoL, and psychological conditions of patients who undergo craniotomy for traumatic brain injuries must be improved and the burdens of their caregivers must be relived. Not only is the care provided by nursing staff equivalent to that offered by neurologists, but in some aspects, it is superior for patients who have undergone craniotomy for traumatic brain injuries and their caregivers during follow-up.

## Introduction

China has a higher number of patients with traumatic brain injuries than most other countries (1). Traumatic brain injury is a major condition that requires several healthcare professionals to provide care in the trauma department (2). In addition, prehospital facilities are insufficient for Chinese emergency care (3). Craniotomy is performed to control intracranial pressure in patients with severe traumatic brain injuries (4). In addition to general postoperative adverse effects (e.g., nausea, vomiting, dizziness), coma, paralysis, mental disabilities, and personality changes are generally reported threats to traumatic brain injuries after craniotomy (2, 5). Craniotomy places patients with traumatic brain injuries at risk of many serious complications, which may affect the outcomes of surgery (6).

Nursing interventions are crucial after neurosurgery and related to patient safety (7). After craniotomy, if nursing care is not sufficiently provided to the patients and the disease conditions of patients are not identified properly and timeously, the possible treatment would be affected, possibly resulting in cognitive impairment or death (8). Improper nursing poses a serious threat after craniotomy in patients with traumatic brain injuries (9). After craniotomy, patients require rehabilitation efforts for the recovery process (10) and neurologists are mostly engaged in the management of post-craniotomy complications (11). However, neurologists are not always available for care after neurosurgery during follow-up (situation of our institute). In addition, in COVID-19 pandemic surgical disciplines have faced serious issues including patients who required surgical care and postoperative follow-up care (12). An important issue is covered as health systems are facing a dire shortage of staff throughout the world. This deserves further scrutiny as to which extent process management can be improved. The findings indicate that the interpersonal skills and communication style of nurses may be more appreciated by patients and caregivers (13). Excellent patient outcomes and high patient satisfaction are two main pillars of the high-value medical care for neurosurgical care of patients (14). In many places, notably the US, post-operative care is typically performed by neurosurgeons with neurologists rarely contributing long-term. Additionally, long-term care varies vastly between settings, largely dependent on infrastructure for patients with a variety of neurodegenerative diseases.

The aim of the retrospective study is to compare the effects of two different types of care (nurse-led and neurologist-led) on various the long-term outcomes (daily living activities (ADL), quality of life (QoL), depression, and anxiety of patients and burden of their caregivers) in patients who have undergone craniotomy due to traumatic brain injuries.

## Materials and methods

### Inclusion criteria

Patients (aged ≥18 years) who underwent craniotomy for traumatic brain injuries were included in the study. Patient underwent decompressive hemicraniectomy, or craniotomy with cranial flap repositioning, or both.

### Exclusion criteria

Patients with incomplete details were excluded from the study.

### Cohort

A total of 109 patients received nurse-led care after craniotomy for 6 months (NL cohort). A total of 121 patients received neurologist-led care for 6 months after craniotomy (GL cohort). In addition to care, all patients received regular treatment prescribed by a consulting neurosurgeon.

## Professional-led post-surgical care

### Neurologist-led follow-up care

Every day a follow-up visits made in ward by neurologist. In the GL cohort medical follow-up is provided by the neurologist alone, and/or by the neurologist assisted by a neurosurgeon and by a physical medicine physician. Neurologist-led care include usual hospital care (counseling of patients and their caregivers regarding threats and adverse effects of surgeries and injuries) and follow-up of patients (routine check-up). During hospital stay minimum 5 min/day provided to patients by neurologists.

### Nurse-led follow-up care

Every day at least two follow-up visits made in ward by staff nurse. In NL cohort medical follow-up is provided by staff nurse oneself. Nurses are minimum postgraduate in nursing care and have minimum 3 years of experiences. Nursing-led care include counseling and education of patients and their caregivers regarding threats and adverse effects of surgeries and injuries. During hospital stay minimum 10 min/day provided to patients by nurses.

After discharge from the hospital, follow-up visits, and telephone consultations with caregivers, and the patients received care from nurses or neurologists. There were no restrictions on telephone consultations with nurses or neurologists for the patients and their caregivers. During the follow-up visit, patients and their caregivers were provided care, counselling, and related instructions for half an hour by a nurse or neurologist. The selection of nurse-led care and neurologist-led care for patients is based on availabilities of professionals in the institutes (in our institutes neurologists are in short to provide follow-up care). There was no crossover between nurse- and neurologist-led aftercare.

## Outcome measures

### Glasgow coma scale (GCS)

GCS score was evaluated before craniotomy. GCS scores were between 3 and 15, with 3 being the worst and 15 being good (15).

### Activities of daily living

ADL was evaluated for six basic activities: toileting, bathing, dressing, eating, moving, and continence of defecation. Each item is scored from 1 to 3 (1 = complete dependence, 2 = partial dependence, and 3 = complete independence). The total score is 18. The higher the score, the higher the ADL (16).

### Quality of life

Physical, emotional, and social functions; somatic pain; general health status; energy; and mental health were used to evaluate QoL. The total score ranged from 0 to 100. The higher the score, the higher the QoL (17).

### The hospital anxiety and depression scale

Seven items were evaluated using the Hospital Anxiety and Depression Scale. Each item has a score ranging from 1 to 3. The total scores for anxiety and depression were 21. The higher the score, the higher the anxiety and depression. A score of >7 was considered anxiety and depression, and a score of >11 was considered definitive anxiety and definitive depression (18).

### Zarit Burden interview

Questionnaires were administered to caregivers to evaluate their psychological burden due to the patient’s condition. It consists of a total of 22 items, and each item is rated on a 0 to 4 scale. The total score was 88, with a higher score indicating a higher burden on caregivers (19).

ADL, QoL, Hospital Anxiety and Depression Scale, and Zarit Burden interview were performed after craniotomy before any professional-led care (nurse-led care or neurologist-led care, BC) and after 6-months of any professional-led care (nurse-led care or neurologist-led care, AC).

### Adverse effects

During the 6-months of any professional-led care, any adverse effects due to surgery and/or treatments were noticed and analyzed.

### Overall satisfaction score

The institute has a print format to evaluate satisfaction. This included emergency care, pre-surgery counseling, surgery, and professional-led post-surgical care. Each item had a score of 0, 1, 2, 3, and 4. 0, completely dissatisfied; 1, dissatisfied; 2, partially satisfied; 3, satisfied; 4, completely satisfied. The total score is 16. The higher the score, the higher the overall satisfaction of patients and caregivers. The overall satisfaction score was evaluated after professional-led postsurgical care for 6 months.

## Statistical analysis

InStat 3.01 (GraphPad Software Inc., San Diego, CA, United States) was used for the statistical analysis. Categorical, continuous normal, and continuous non-normal variables are presented as frequencies with percentages in parentheses, mean ± standard deviation (SD), and medians with Q3–Q1 in parentheses, respectively. All results were considered significant if the *p*-value was less than 0.05. The Kolmogorov–Smirnov method was adopted to check the normality of continuous variables. If at least one column failed in the normality test with *p* < 0.05, a nonparametric test (Mann–Whitney test or Kruskal-Wallis’ test) was performed; otherwise, parametric tests (paired *t*-test or unpaired *t*-test) were performed for continuous variables. Fisher’s exact test or chi-square test (*χ*^2^-test) was used for the statistical analysis of categorical variables. Dunn’s multiple comparison test was used for *post hoc* analysis. The Quartile Calculator^1^ was used to calculate the Q3 (third quartile) and Q1 (first quartile) values. All results were considered significant if the *p*-value was less than 0.05.

## Results

### Study population

**Figure 1:**
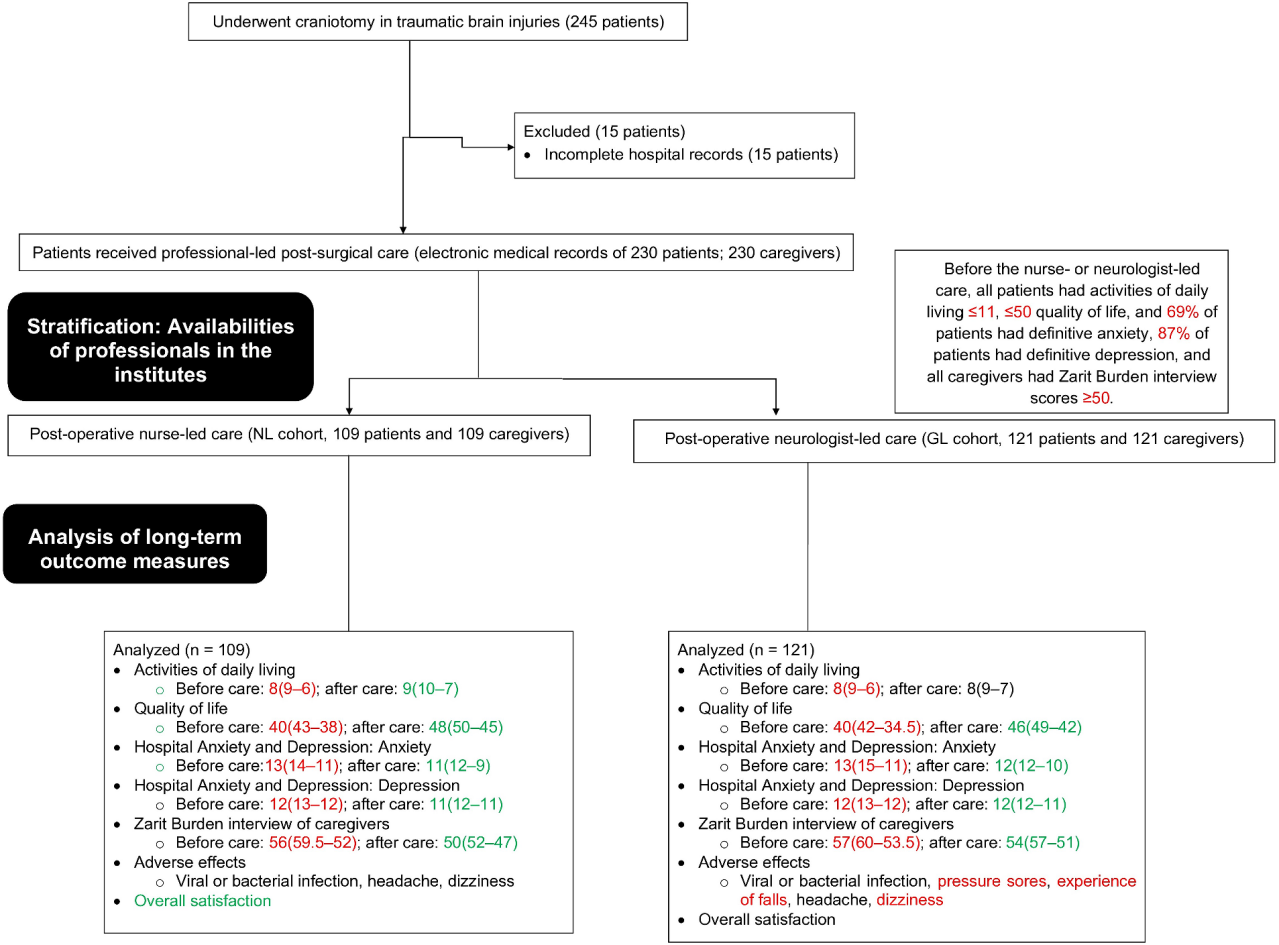
The study flow chart. Red color indicates worse results. Green color indicates favorable results. Black color indicates not favorable but not worse results.

For retrospective study electronic medical records of patients were searched to extract data. From January 18, 2019, to February 20, 2023, 245 patients (age ≥ 18 years) underwent craniotomy for traumatic brain injuries at the Shanxi Bethune Hospital, Taiyuan, Shanxi, China and the referring institutes. Data from 15 patients were incomplete in hospital records. Therefore, these patients were excluded from this study. ADL, QoL, the Hospital Anxiety and Depression Scale of patients, Zarit Burden interview of caregivers, and the overall satisfaction score of patients and caregivers for a total of 230 patients and their caregivers were included in the study. The flowchart of the study is shown in Figure 1.

### Demographic and clinical conditions

The number of male patients was higher than that of female patients. Most patients had motor vehicle accidents due to traumatic brain injuries. All patients had a GCS score < 10 before the operation. Most of the patients underwent surgery on the dominant side. At BC, all patients had an ADL of 11 or fewer, all patients had a QoL of 50 or less, and most of them had definitive anxiety (69%) and depression (87%). Age, sex, reason for traumatic brain injuries, Glasgow Coma Scale score before the operation, surgery side, ADL, QoL, anxiety, and depression of patients were statistically similar between cohorts at BC (*p* > 0.05, Table 1).

**Table 1:** Demographic and clinical conditions of patients after craniotomy for traumatic brain injuries before professional-led post-surgical care.

Parameters		Cohorts		Comparisons between cohorts			
		NL	GL				
Professional for post-surgical care		Nurse	Neurologist				
Numbers of patients		109	121	*p*-value	df	Test value	95% CI
Age (years)		36 (44.5–31)	41 (46–33)	0.1675 (Mann–Whitney test)	N/A	5898.5	N/A
Gender	Male	75 (69)	70 (58)	0.1136 (*χ*^2^-test with Yates correction)	1	2.503	0.9539 to 1.753
	Female	34 (31)	51 (42)				
*Reason for traumatic brain injuries*							
Motor vehicle accident		89 (82)	95 (78)	0.8721 (*χ*^2^-test for independence)	3	0.7048	N/A
Fall		8 (7)	12 (10)				
War or training		7 (6)	7 (6)				
Violence		5 (5)	7 (6)				
^@^Glasgow Coma Scale before the operation		7 (8–7)	7 (8–7)	0.4929 (Mann–Whitney test)	N/A	6252.5	N/A
*Surgery side*							
Dominant side		65 (60)	65 (54)	0.4788 (*χ*^2^-test for independence)	2	1.473	N/A
Non-dominant side		35 (32)	48 (40)				
Bilateral		9 (8)	8 (6)				
ADL		8 (9–6)	8 (9–6)	0.8681 (Mann–Whitney test)	N/A	6510.5	N/A
QoL		40 (43–38)	40 (42–34.5)	0.3317 (Mann–Whitney test)	N/A	6,105	N/A
Anxiety scale	≤7	0 (0)	0 (0)	0.8075 (*χ*^2^-test with Yates correction)	1	0.0593	0.7935 to 1.414
	8–11	35 (32)	36 (30)				
	>11	74 (68)	85 (70)				
	Value	13 (14–11)	13 (15–11)	0.2283 (Mann–Whitney test)	N/A	5931.5	N/A
Depression scale	≤7	0 (0)	0 (0)	0.2291 (Mann–Whitney test)	N/A	5993.5	N/A
	8–11	10 (9)	21 (17)				
	>11	99 (91)	100 (83)				
	Value	12 (13–12)	12 (13–12)				

Categorical and continuous normal or ordinal variables are depicted as frequencies with percentages and medians with Q3–Q1 in parenthesis, respectively.

All results are considered significant if the *p*-value is less than 0.05.

CI: Confidence interval (using the approximation of Katz. for categorical variables), N/A: Not applicable, df: Degree of freedom.

Test value (Mann–Whitney U-statistics for Mann–Whitney test, χ^2^-value for χ^2^-test).

^@^Values was between 3 and 15, 3 being the worst and 15 being good.

ADL: Activities of daily living (total score is 18. Higher the score, the higher is ADL).

QoL: Quality of life (the total score range was from 0 to 100. The higher the score higher is QoL).

Anxiety scale (total score was 21. The higher the score higher is anxiety).

Depression scale (total score was 21. The higher the score higher is depression).

≤ 7: No anxiety/ depression.

8–11: moderate anxiety/ depression.

11 >: definite anxiety/depression.

All caregivers had Zarit Burden Interview scores of 50 or more at the BC. One patient had a caregiver. Most of the caregivers were spouses. The details of comorbidities of caregivers were not available in the hospital records. Age, sex, education, and Zarit Burden interview scores of caregivers were statistically the same between both cohorts (*p* > 0.05, Table 2).

**Table 2:** Available demographic and clinical conditions of caregivers of patients after craniotomy for traumatic brain injuries before professional-led post-surgical care of patients.

Parameters		Cohorts		Comparisons between cohorts			
		NL	GL				
Professional for post-surgical care of patients		Nurse	Neurologist				
Numbers of caregivers		109	121	*p*-value	df	Test value	95% CI
Age (years)		36 (45–31)	41 (45–31)	0.5791 (Mann–Whitney test)	N/A	6314.5	N/A
Gender	Male	59 (54)	60 (50)	0.5781 (*χ*^2^-test with Yates correction)	1	0.3093	0.8369 to 1.448
	Female	50 (46)	61 (50)				
Education	Primitive	10 (9)	12 (10)	0.9776 (*χ*^2^-test for independence)	3	0.1998	N/A
	Below higher secondary	63 (58)	72 (59)				
	Above higher secondary but not graduate	25 (23)	25 (21)				
	Graduate or above or professional course	11 (10)	12 (10)				
Zarit Burden interview score		56 (59.5–52)	57 (60–53.5)	0.3194 (Mann–Whitney test)	N/A	6092.5	N/A

Categorical and continuous ordinal variables are depicted as frequencies with percentages in parenthesis and medians with Q3–Q1 in parenthesis, respectively.

All results are considered significant if the *p*-value is less than 0.05.

CI: Confidence interval (using the approximation of Katz. for categorical variables), N/A: Not applicable, df: Degree of freedom.

Test value (Mann–Whitney U-statistics for Mann–Whitney test, χ^2^-value for χ^2^-test).

Zarit Burden interview score (Total score is 88, higher score indicates higher burden on).

### Outcome measures

Nurse-led post-surgical care was associated with improved ADL of patients with AC condition as compared to their BC condition and also compared to those of the GL cohort in the AC condition. Neurologist-led care was not successful in improving ADL at AC condition compared with their BC condition. Nurse-led care and neurologist-led care were associated with improved QoL, relieved anxiety and depression in patients, and relieved the burden on caregivers of patients at AC condition compared to their BC conditions. In addition, patients in the NL cohort had a better QoL and less anxiety than those in the GL cohort in the AC condition. Caregivers of patients in the NL cohort had fewer burdens than those of caregivers of patients in the GL cohort in the AC condition. The overall satisfaction scores of patients and their caregivers were higher for nurses than that scores for neurologists. The details of the outcome measure of patients and their caregivers after craniotomy for traumatic brain injuries before and after professional-led post-surgical care are presented in Table 3.

**Table 3:** The outcome measures of patients and their caregivers after craniotomy for traumatic brain injuries before and after professional-led post-surgical care.

Parameters	Cohorts								Comparisons between cohorts at AC	
	NL				GL					
Professional for post-surgical care	Nurse				Neurologist					
Numbers of patients	109	109	Comparisons between BC and AC		121	121	Comparisons between BC and AC			
Numbers of caregivers	109	109			121	121				
Level	BC	AC	*p*-value	Kruskal-Wallis’ test-statistics	BC	AC	*p*-value	Kruskal-Wallis’ test-statistics	*p*-value	Kruskal-Wallis’ test-statistics
ADL of patients	8 (9–6)	9 (10–7)	<0.001	26.551	8 (9–6)	8 (9–7)	0.1304	4.074	<0.01	21.312
QoL of patients	40 (43–38)	48 (50–45)	<0.001	140.42	40 (42–34.5)	46 (49–42)	<0.001	96.504	<0.05	121.13
Anxiety scale of patients	13 (14–11)	11 (12–9)	<0.001	83.463	13 (15–11)	12 (12–10)	<0.001	39.052	<0.01	61.378
Depression scale of patients	12 (13–12)	11 (12–11)	<0.001	92.027	12 (13–12)	12 (12–11)	<0.001	70.443	0.1118	5,802
Zarit Burden interview score of caregivers	56 (59.5–52)	50 (52–47)	<0.001	110.76	57 (60–53.5)	54 (57–51)	<0.001	21.524	<0.001	86.834
Overall satisfaction score of patients and their caregivers	N/A	11 (12–10)	N/A	N/A	N/A	9 (10–9)	N/A	N/A	<0.0001	4,354

Variables are depicted as medians with Q3–Q1 in parenthesis.

Mann–Whitney test or Kruskal-Wallis’ test was used for statistical analysis. Dunn’s Multiple Comparisons test was used for *post hoc* analysis.

All results are considered significant if the *p*-value is less than 0.05.

BC: Before any professional (nurse or neurologist)-led care, AC: After 6 months of any professional (nurse or neurologist)-led care, N/A: Not applicable.

ADL: Activities of daily living (Total score is 18. Higher the score, the higher is ADL).

QoL: Quality of life (The total score range was from 0 to 100. The higher the score higher is QoL).

Anxiety scale (Total score was 21. The higher the score higher is anxiety).

Depression scale (Total score was 21. The higher the score higher is depression).

Zarit Burden interview score (Total score is 88, a higher score indicates a higher burden on caregivers).

Overall satisfaction score (Total score is 16. The higher the score, the higher the overall satisfaction of patients and caregivers).

#### Anxiety and depression

Nurse-led care and neurologist-led care both successfully decreased anxiety and depression from definite anxiety (score > 11) to moderate anxiety (score 8–11) in the AC condition compared with their BC conditions. In addition, nurse-led care was more successful in decreasing the anxiety of patients with definite anxiety (score > 11) to moderate anxiety (score 8–11) in the AC condition than those did by neurologist-led care. Nurse-led care or neurologist-led care failed to complete reduce anxiety or depression in patients (score ≤ 7) at the AC condition. The details of the hospital anxiety and depression scales are presented in Table 4.

**Table 4:** The details of the hospital anxiety and depression scales.

Scale	Cohorts												Comparisons between cohorts			
	NL						GL									
Professional for post-surgical care	Nurse						Neurologist									
Level	BC	AC	*p*-value	df	Test value	95% CI	BC	AC	*p*-value	df	Test value	95% CI	*p*-value	df	Test value	95% CI
Numbers of patients	109	109					121	121								
**Anxiety**																
≤7	0 (0)	1 (1)	<0.0001 (*χ*^2^-test for independence)	2	21.713	N/A	0 (0)	0 (0)	0.045 (*χ*^2^-test with Yates correction)	1	4.018	0.5553 to 0.9892	0.0059 (*χ*^2^-test for independence)	2	10.251	N/A
8–11	35 (32)	68 (62)					36 (30)	52 (43)								
>11	74 (68)	40 (37)					85 (70)	69 (57)								
**Depression**																
≤7	0 (0)	0 (0)	<0.0001 (Fisher’s exact test)	N/A	0.2228	0.1243 to 0.3995	0 (0)	0 (0)	<0.0001(*χ*^2^-test with Yates correction)	1	0.45	0.3062 to 0.6613	0.0828 (Fisher’s exact test)	N/A	0.6484	0.3820 to 1.101
8–11	10 (9)	58 (53)					21 (17)	56 (46)								
>11	99 (91)	51 (47)					100 (83)	65 (54)								

Variables are depicted as frequencies with percentages in parentheses.

All results are considered significant if the *p*-value is less than 0.05.

≤ 7: No anxiety/ depression.

8–11: moderate anxiety/ depression.

11 >: definite anxiety/depression.

Test value (χ^2^-value for χ^2^-test, relative risk for Fisher’s exact test).

BC: Before any professional (nurse or neurologist)-led care, AC: After 6-months of any professional (nurse or neurologist)-led care, df: Degree of freedom, CI: Confidence interval (using the approximation of Katz.), N/A: Not applicable.

### Adverse effects

None of the patients died during the study. Headache was commonly reported in patients in both cohorts. Pressure sores, dizziness, and experience of falls were reported to be higher in patients in the GL cohort than in those in the NL cohort. The total numbers of adverse events and adverse effects per patient were higher in patients in the GL cohort than those in the NL cohort. The details of adverse effects during the six months of any professional-led care and follow-up period (extra six months) are reported in Table 5.

**Table 5:** The details of adverse effects during the six months of any professional-led care and follow-up period.

Effect	Cohorts		Comparisons between cohorts			
	NL	GL				
Professional for post-surgical care	Nurse	Neurologist				
Numbers of patients	109	121	*p*-value	df	Test value	95% CI
Viral or bacterial infection	3 (3)	4 (3)	0.9999 (Fisher’s exact test)	N/A	0.9016	0.3790 to 2.145
Pressure sores	2 (2)	11 (9)^*^	0.0211 (Fisher’s exact test)	N/A	0.312	0.08655 to 1.125
Rebleeding	1 (1)	2 (2)	0.9999 (Fisher’s exact test)	N/A	0.7006	0.1405 to 3.493
Central hyperthermia	1 (1)	1 (1)	0.9999 (Fisher’s exact test)	N/A	1.056	0.2621 to 4.251
Experience of falls	3 (3)	10 (8)	0.0889 (Fisher’s exact test)	N/A	0.4724	0.1734 to 1.287
Hypertension	2 (2)	4 (3)	0.686 (Fisher’s exact test)	N/A	0.6978	0.2231 to 2.182
Nausea	3 (3)	4 (3)	0.9999 (Fisher’s exact test)	N/A	0.9016	0.3790 to 2.145
Headache	15 (14)	21 (17)	0.4737 (Fisher’s exact test)	N/A	0.8599	0.5690 to 1.3
Dizziness	5 (5)	15 (12)	0.0585 (Fisher’s exact test)	N/A	0.5048	0.2334 to 1.092
Total numbers of adverse events	30	57^*^	0.0035 (*χ*^2^-test with Yates correction)	1	8.538	0.4509 to 0.8640
Adverse effect/ patient	0.28	0.47	N/A	N/A	N/A	N/A

Variables are depicted as frequencies with percentages in parentheses.

Patients had one or more adverse effect(s).

All results are considered significant if the *p*-value is less than 0.05.

CI: Confidence interval (using the approximation of Katz), N/A: Not applicable, df: Degree of freedom.

Test value (relative risk for Fisher’s exact test, χ^2^-value for χ^2^-test).

^*^Significant adverse effects were reported in patients of the GL cohort than those of the NL cohort.

Adverse effects after craniotomy for traumatic brain injuries before professional-led post-surgical care were not included in the above analysis.

## Discussion

At BC condition, all patients had ≤11 ADL and ≤ 50 QoL, and most patients had definitive anxiety and depression. In addition, at BC condition all caregivers of patients had Zarit Burden interview scores ≥50. After craniotomy for traumatic brain injuries, the patients had worse ADL, QoL, anxiety, and depression. The outcome results of the patients after craniotomy for traumatic brain injuries are consistent with those of a case–control study (9), a propensity matching study (2), an observational study (17), and a pilot study (7). ADL and QoL of patients after craniotomy for traumatic brain injuries must be improved and anxiety and depression of patients after craniotomy for traumatic brain injuries must be relieved. In addition, after craniotomy for traumatic brain injuries, the burden on caregivers of patients must be relieved.

Nurse-led care improved ADL and QoL and relieved anxiety and depression in patients with AC conditions as compared to their BC conditions and those of patients of the GL under AC conditions. The results of the NL cohort were consistent with those of a case–control study (9) and a trial on multiple sclerosis (13). In addition, nurse-led care relieved the burden on caregivers of patients with AC conditions compared to the BC conditions and those of caregivers of patients with the GL cohort under AC conditions. Nurse-led care and neurologist-led care are associated with the improvement of ADL and QoL, relief of anxiety and depression in patients after craniotomy for traumatic brain injuries, and relief of burden on caregivers. Moreover, nurse-led care is superior to neurologist-led care in the follow-up of patients after craniotomy for traumatic brain injuries, regarding the improvement of ADL, QoL, and psychological conditions of patients and burdens of their caregivers. Frequent visits of nurses to patients and caregivers than those of neurologists are the reasons for these results.

Patients in the GL cohort reported pressure sores, dizziness, experience of falls, higher numbers of total adverse effects, and higher adverse effects per patient during six months of care and a follow-up period than those in the NL cohort. Neurologists are not always available for care after neurosurgery during follow-up to provide mobility to patients (situation of our institute). This would lead to a high risk of pressure sores, falls while walking, and dizziness in patients of the GL cohorts (20). Nurse-led care during follow-up decreases adverse effects after craniotomy for traumatic brain injuries compared with neurologist-led follow-up care.

The overall satisfaction scores of patients and their caregivers were higher for nurses than for neurologists. Compared with neurologists, nurses provide more time to patients and their caregivers. The results of the overall satisfaction scores of patients and their caregivers of the current study are consistent with a trial on multiple sclerosis (13). In addition, nurses have better communication skills and require fewer waiting times than neurologists (21). Nurses provide advanced skills to various complex medical conditions, with potential to enhance the health, cognitive functioning, and quality of life (22). Nurses can also develop interpersonal relationships. This would increase the satisfaction of the patients and caregivers.

These interpretations of the results cannot be extrapolated as an indication of the best treatment for the recovery of these patients, which must include multidisciplinary management, where in addition to neurological doctors and nurses, many other professionals participate, such as neuropsychologists, rehabilitators, physiotherapists, speech therapists, etc. Each of them will contribute in each area to try to maximize the possibilities of functional recovery. However, it is obvious that the local context may prevent having all the resources available and that these are optimized and, in this situation, directed in the best possible way.

Both nurse-led and neurologist-led care were unsuccessful in completely removing anxiety or depression in patients (score ≤ 7) at AC. This suggests that additional treatment interventions may be needed to address these (anxiety and depression) issues of patients.

In the current study research outcome measures such as ADL, QoL, the Hospital Anxiety and Depression Scale of patients, and Zarit Burden interview of caregivers, as well as the overall satisfaction score of patients and caregivers were evaluated. The objectives of study were to evaluate long-term outcome measures of patients who have undergone craniotomy due to traumatic brain injuries. These (ADL, QoL, the Hospital Anxiety and Depression Scale of patients, and Zarit Burden interview of caregivers) are good predictors of long-term outcome measures (2).

The study claims that this article is a retrospective study, and it is well known that retrospective studies look at past data to conclude the present. In the daily work of hospitals, many aspects are not routinely evaluated, such as ADL and Zarit Burden interviews. In general practice, these indicators mentioned in the article are obtained through retrospective research. The possible justification for the same is that in the daily work of our hospital many aspects, such as ADL and Zarit Burden interviews are routinely evaluated.

The compilation of the comparative studies on professional-led post-surgical care after craniotomy (neurosurgeries) for traumatic brain injuries used in the current study for re-iteration of research findings (current research or past researches) is presented in Table 6.

**Table 6:** Comparative studies on professional-led post-surgical care after craniotomy (neurosurgeries) for traumatic brain injuries.

Study	Published year	Ethnicity	Study population	Sample size (N; patients)	Age range
Pilot study, Johnson et al. (7)	2021	North America	Patients with indwelling shunts	8 patients	14–18 years
Case–control study, Jing et al. (9)	2022	Chinese	Patients undergoing neurosurgery	120 patients	18–75 years
Scoping review, Sveen et al. (10)	2022	World population	Patients faced traumatic brain injury	425 articles	Adult (range is not specified)
Randomized trial, Smyth et al. (13)	2022	Canadian	People with Multiple Sclerosis	220 patients	47.47 ± 11.02
Retrospective study, Young et al. (14)	2020	North America	Patients admitted to the neurosciences intensive care unit following a craniotomy	314 patients	40–70 years
Observational study, Zhao and Wang, (17)	2023	Chinese	Patients undergoing craniocerebral injury surgery	105 patients	Adult (range is not specified)
Observational study, Hong et al., (20)	2021	Chinese	Intracerebral hemorrhage patients	72 patients	60–85 years

In the study the results obtained in the late care of patients undergoing surgery for a traumatic brain injury are compared depending on whether these cares were provided by neurologists or nursing staff. The reason for raising this dilemma and this analysis seems to be the lack of medical personnel in the area of origin of the study. However, there are few limitations of this study, for example, its retrospective design and lack of randomized trials, with inherent limitations in obtaining information and biases in its analysis. The study has small cohorts which limits generalizability. Future research should consider incorporating more objective measures and providing more detailed information on adverse effects.

## Conclusion

The activities of daily living and quality of life of patients after craniotomy for traumatic brain injuries must be improved, and anxiety and depression of patients after craniotomy for traumatic brain injuries must be relieved. Burden of caregivers of patients must be relieved after craniotomy for traumatic brain injuries. Nurse-led care and neurologist-led care are associated with improvement in quality of life, relief of anxiety and depression in patients after craniotomy for traumatic brain injuries, and relief of burden on caregivers of patients. Nurse-led care is superior to neurologist-led care in six months of the follow-up of patients after craniotomy for traumatic brain injuries, regarding the improvement of activities of daily living, quality of life, and psychological conditions of patients and burdens of their caregivers. Nurse-led care during follow-up decreases adverse effects after craniotomy for traumatic brain injuries compared with neurologist-led follow-up care. Patients and their caregivers may have higher satisfaction for nurses than neurologists with follow-up care after craniotomy for traumatic brain injuries. So is the consideration that patients after craniotomy for traumatic brain injuries have of nurse-led care. This study provides additional context and support for the benefits of nurse-led care after craniotomy in traumatic brain injuries.

## Data availability statement

The original contributions presented in the study are included in the article/supplementary material, further inquiries can be directed to the corresponding author.

## Ethics statement

The study protocol was designed and approved by the human ethics committee of the Shanxi Bethune Hospital and the Chinese Neurotrauma Scholar Association (Approval number 2019YJ18 dated January 11, 2019). The study follows the laws of China and the v2008 Declarations of the Helsinki. The studies were conducted in accordance with the local legislation and institutional requirements. The participants provided their written informed consent to participate in this study.

## Author contributions

JW: Conceptualization, Methodology, Software, Visualization, Writing – original draft, Writing – review & editing. ZW: Methodology, Project administration, Resources, Software, Validation, Visualization, Writing – original draft, Writing – review & editing. SS: Methodology, Resources, Software, Visualization, Writing – original draft, Writing – review & editing. JR: Data curation, Investigation, Methodology, Software, Supervision, Visualization, Writing – original draft, Writing – review & editing. XR: Conceptualization, Data curation, Formal analysis, Funding acquisition, Methodology, Writing – original draft, Writing – review & editing.

## Footnotes

^1^ https://www.calculatorsoup.com/calculators/statistics/quartile-calculator.php
